# Optimal distribution grid allocation of reactive power with a focus on the particle swarm optimization technique and voltage stability

**DOI:** 10.1038/s41598-024-61412-9

**Published:** 2024-05-13

**Authors:** Oriza Candra, Mohammed I. Alghamdi, Ali Thaeer Hammid, José Ricardo Nuñez Alvarez, Olga V. Staroverova, Ahmed Hussien Alawadi, Haydar Abdulameer Marhoon, M. Mehdi Shafieezadeh

**Affiliations:** 1 Department Teknik Elektro and Electrical Power Engineering Research Group, Universitas Negeri Padang, Padang, Indonesia; 2 Computer Science Department, Al-Baha University, Al-Baha City, Kingdom of Saudi Arabia; 3 Technical College of Engineering, Al-Bayan University, Baghdad, Iraq; 4 Energy Department, Universidad de la Costa, Barranquilla, Colombia; 5 Department of State and Municipal Finance, Plekhanov Russian University of Economics, Stremyanny Lane, 36, Moscow, Russian Federation 117997; 6 The Islamic University, Najaf, Iraq; 7 Information and Communication Technology Research Group, Scientific Research Center, Al-Ayen University, Nasiriyah, Thi-Qar Iraq; 8 College of Computer Sciences and Information Technology, University of Kerbala, Karbala, Iraq; 9 Department of Chemical Engineering, Islamic Azad University, Shiraz, Iran; 10 Department of Electronics Engineering, College of Engineering, University of Diyala, Baqubah, Diyala 32001 Iraq; 11 Sumerian Scriptum Synthesis Publisher, Baqubah, Diyala Province 32001 Iraq

**Keywords:** Reactive power, Voltage stability, Ancillary service, PSO algorithm, Engineering, Electrical and electronic engineering

## Abstract

A structured approach to managing reactive power is imperative within the context of power systems. Among the restructuring initiatives in the electrical sector, power systems have undergone delineation into three principal categories: generation, transmission, and distribution entities, each of which is overseen by an independent system operator. Notably, active power emerges as the predominant commodity transacted within the electrical market, with the autonomous grid operator assuming the responsibility of ensuring conducive conditions for the execution of energy contracts across the transmission infrastructure. Ancillary services, comprising essential frameworks for energy generation and delivery to end-users, encompass reactive power services pivotal in the regulation of bus voltage. Of particular significance among the array of ancillary services requisite in a competitive market milieu is the provision of adequate reactive power to uphold grid safety and voltage stability. A salient impediment to the realization of energy contracts lies in the inadequacy of reactive power within the grid, which poses potential risks to its operational safety and voltage equilibrium. The optimal allocation of the reactive power load is predicated upon presumptions of consistent outcomes within the active power market. Under this conceptual framework, generators are afforded continual compensation for the provision of reactive power indispensable for sustaining their active energy production endeavors.

## Introduction

Reactive power dispatch constitutes a fundamental component of power system operations, primarily tasked with the regulation of voltage stability and attenuation of line losses^1–5^. Particularly within distribution systems and microgrids, where the resistance-to-reactance ratio surpasses that of transmission systems, the implementation of localized reactive power compensation holds substantial potential in diminishing power losses and, consequently, operational expenditures^6–10^. In pursuit of local reactive power provision, numerous scholars have investigated the optimal allocation and operational strategies concerning reactive power compensation devices within distribution systems^11–15^. The study presented in reference^16^ introduces a novel slack bus independent loss allocation methodology tailored for the bilateral market setting. Within this framework, the generator assumes responsibility for supplying power to both loads and their accompanying losses, thereby distributing the total load and its associated losses among all generators interconnected within the network. In contrast, reference^17^ outlines an alternative loss allocation technique wherein generators and loads are depicted as current injections and impedances, respectively. Employing principles derived from circuit theory and the Aumann–Shapley method, this approach offers a refined mechanism for loss allocation^18–22^. Furthermore, the paper proposes a fresh pricing model applicable to bilateral, pool, and reserve markets, leveraging the optimal power flow (OPF) paradigm^23–27^.

In reference^28^, researchers employed an ant colony optimization (ACO) algorithm to optimize the overall cost of electrical energy generated by distribution companies (Discos) and distributed generators (DGs) within the framework of the daily voltage/var control (VVC) problem. Conversely, reference^6^ presented a novel fuzzy price-based compensation methodology designed to address the daily VVC conundrum in distribution systems amid the presence of DGs. Furthermore, in reference^29^, a pioneering optimization algorithm centered on a chaotic improved honey bee mating optimization (CIHBMO) approach was implemented. This algorithm facilitates the determination of critical control variables for the subsequent day, encompassing the active and reactive power of DG units, reactive power settings of capacitors, and tap positions of transformers. Similarly, reference^30^ introduces a fuzzy adaptive chaotic particle swarm optimization (FACPSO) technique to address the multiobjective optimal operation management of distribution networks inclusive of fuel-cell power plants. In^31–34^, a methodology for minimizing active power losses and microgeneration shedding was proposed. This approach aims to achieve optimized and coordinated voltage support within distribution networks characterized by significant integration of DGs and microgrids. Finally, in reference^35–37^, an innovative approach combining an analytic hierarchy process (AHP) strategy with a binary ant colony optimization (BACO) algorithm was employed to resolve the multiobjective daily VVC problem encountered in distribution systems. The slime mold algorithm (SMA) is a nature-inspired optimization algorithm inspired by the behavior of slime molds, which are simple organisms capable of complex behaviors such as pathfinding and optimization. This algorithm is particularly useful for solving optimization problems, inspired by the ability of slime molds to find efficient routes in complex environments. In^38–41^, a sliding mode algorithm (SMA)) search strategy was proposed for solving the optimal power flow (OPF) and reactive power dispatch problem. The Runge‒Kutta method is a numerical technique used for solving ordinary differential equations (ODEs) and systems of ODEs and was used for analysis and load power flow dispatch in^42^. HHO is a nature-inspired optimization algorithm in which each potential solution is represented as a bird in a population, and these birds collaborate to find the optimal solution to a given optimization problem. The algorithm simulates the hunting behavior of Harris's hawks, including exploration, exploitation, and communication among individuals.^43–45^ used the HHO method to optimize the reactive and active power flow to stabilize the voltage.

Given the significantly greater share of active power generation costs compared to reactive power generation costs, the attention devoted to the behavior of the latter in the reactive market tends to be relatively diminished. However, in fuzzy references^46,47^, a model is introduced that aims to simultaneously minimize the total cost associated with active and reactive power generation. This research endeavors to devise a model for optimizing the distribution of reactive power, taking into consideration not only the maintenance of voltage levels within permissible thresholds but also the preservation of voltage stability throughout operational phases^48–51^.

In this study, a hybrid approach incorporating the generator cross-section auction model^52–55^ and static compensator cost functions is employed. Moreover, the model encompasses the calculation of both the active and reactive power outputs of generators, along with the determination of reactive power levels for compensators and the associated cost of transformer tap changers. It is noteworthy that this methodology assumes constant outcomes in the active market, with considerations also given to the temporal evolution and fluctuation of grid load. Notably, this method neglects the slack bus generator from certain considerations^56–58^. The motivation for using particle swarm optimization (PSO) for reactive power dispatch (RPD) in power systems arises from several factors. Complexity of the Problem: Reactive power dispatch is a complex optimization problem involving nonlinear equations, multiple constraints, and a large solution space. PSO, as a metaheuristic optimization algorithm, offers a robust and efficient way to search through this complex solution space to find near-optimal solutions. Multidimensional Search Space: Reactive power dispatch involves adjusting the settings of various reactive power devices, such as generators, capacitors, and transformers^59–62^. The ability of PSO to explore multidimensional search spaces makes it suitable for finding optimal or near-optimal configurations of these devices to improve system performance. Real-Time Application: PSO is known for its computational efficiency, which is crucial for real-time or near-real-time applications in power systems. Reactive power dispatch needs to be performed rapidly to maintain system stability and reliability, and the fast convergence properties of PSO make it suitable for such applications. Adaptability and Tunability: PSO is highly adaptable and adaptable to specific problem characteristics and requirements. This flexibility allows researchers and engineers to customize the PSO algorithm to suit the unique aspects of reactive power dispatch problems, such as incorporating penalty functions or adjusting inertia weights^63–67^.

The subsequent sections of the paper are structured as follows:

- Section “Voltage stability margin index” discusses the voltage stability margin and examines its correlation with the power grid load and stability.
- Section “Optimal reactive power planning model” elucidates the planning model devised for reactive power, outlining its fundamental principles and methodologies.
- Sections “Problem objective function” and “Inequality constraints” are dedicated to identifying the objective function and constraints inherent in the proposed model, respectively.
- Section “Using particle swarm optimization to solve the second mode optimization” provides an in-depth explanation of the Particle Swarm Optimization (PSO) algorithm utilized for optimizing the model, along with the presented results.
- The concluding section encapsulates the findings and implications drawn from the study, offering insights and potential avenues for future research.

## Voltage stability margin index

**Figure 1:**
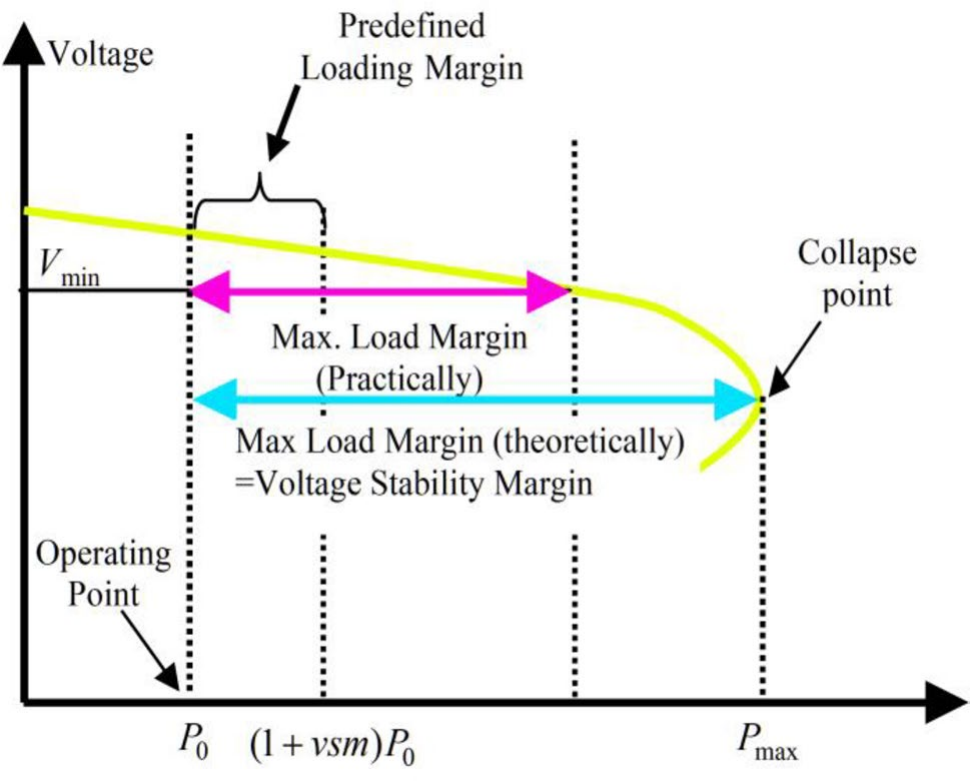
Voltage diagram in terms of the power grid load and voltage stability.

There is currently a strong economic incentive to utilize the entire capacity of the electricity system^68–71^. The independent grid operator must be aware of the stability status and the distance to the edge of grid instability since rising power generation and demand are pushing the system to the point of instability^29–31,34^. It is a valuable indicator for making judgments since it can quantify the distance from the absolute location of the system's instability to the grid operator. The grid load limit index, which can be used as a gauge of the maximum voltage stability margin, is the sole indicator that possesses this property^72–75^. The voltage diagram is shown in Fig. 1 as a power diagram, where $vsm$ denotes the system's loading parameter. This graph demonstrates that even when the system's overall load is increased by a factor of $vsmP_{0}$, voltage collapse does not occur.

## Optimal reactive power planning model

This section presents a model that integrates operational reactive power management considerations. Voltage stability, a critical aspect influenced by various factors, including the spatial arrangement and magnitude of active power generation across the grid, the configuration of the distribution system, and the placement of reactive power supply assets^40–42^, is of paramount importance. In light of these multifaceted determinants, this section endeavors to delineate a reactive power distribution system that not only prioritizes voltage stability during operational phases but also ensures the preservation of voltage amplitude within acceptable limits.

## Problem objective function

With increasing system load, $Q_{GENi}$ and $Q_{SHi}$ in the equation below indicate the reactive energy obtained from the i-th generator and the i-th static compensator, respectively. $\Delta P_{GEN}^{slack}$ is the additional power obtained from the slack bus generator to increase the load and offset losses, and $\Delta T_{Ti}$ is the modification to the *ith* transformer's conversion ratio. $\gamma_{Ti}$ The cost of reactive electricity generated by the i-th generator is determined by the *i*-th generator's auction price function and this statement. The cost of acquiring static reactive power compensation for the grid is included in the second expression to the right of this equation, and the cost of changing the taps on transformers is included in the third expression. The final sentence also includes the price of the electricity drawn from the slag bus generator.

1 $$\begin{aligned}&MinF(Q_{GEN} ,Q_{SH} ,\Delta P_{GEN}^{slack} ,\Delta T) = \sum\limits_{i = 1}^{{N_{GEN} }} {\lambda_{GENi} (} Q_{GENi} ) \hfill \\& \quad + \sum\limits_{i = 1}^{{N_{SH} }} {C_{SHi} (} Q_{SHi} ) + \sum\limits_{i = 1}^{{N_{T} }} {C_{Ti} (} \Delta T_{Ti} ) + C(\Delta P_{GEN}^{slack} ) \hfill \\ \end{aligned}$$

## Equality constraints

The equality of the active and reactive power produced with consumption is one of these restrictions. In this instance, it is presumable that generators are the only source of the necessary reactive power. Both the zero voltage angle of the slack bus and the stability of the voltage size of the bus with the generator are considered equal constraints. The active power equalization limits only consider the active power produced by the slack bus generator when the system experiences a rapid increase in load during operation. The reactive power equalizer restrictions also include static compensators in addition to generators. In this case, the stability of the active power produced by the generators, with the exception of the slack bus generator, is regarded as an equality requirement.

## Inequality constraints

**Figure 2:**
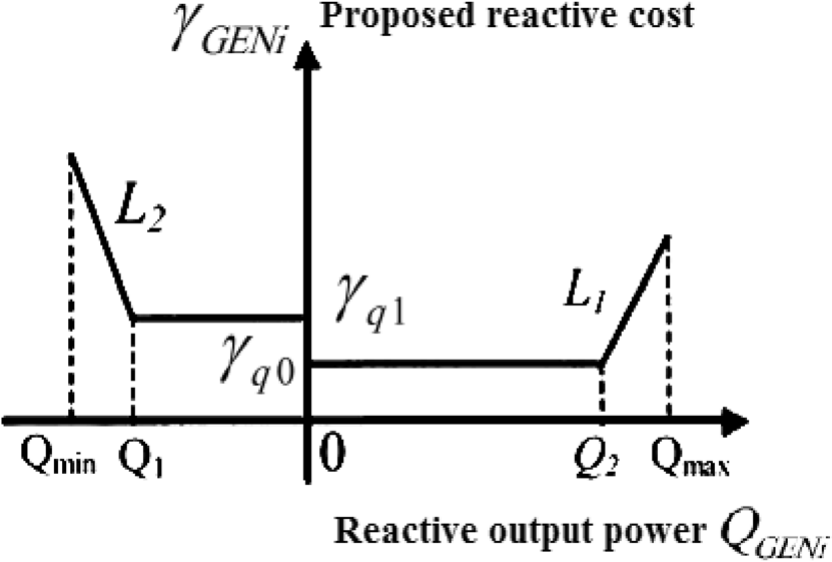
Generic generator auction curve for reactive power.

Static compensators, generator output power, and reactive power generation or absorption capacity are used as inequality restrictions. The permitted range of changes in the transformer Tap, the temperature limit of the transmission lines, and the size and angle of the bus voltage are further inequality restrictions. The reactive power auction for a generator's entire curve is shown in Fig. 2. Figure $\gamma_{GENi}$ shows the reactive power obtained from the i-th generator cost.

A generator's maximum reactive power output is determined by the quantity of active power generation, following its operating capability curve. Taking the Slack Bass generator used in this project as an example, the auction function is as follows:

2 $$\gamma_{GEN2}(Q_{GEN2} ) = \left\{ {\begin{array}{*{20}c} { - 1.5Q_{GEN2} - 0.2} & { - 0.2 \le Q_{GEN2} < 0.12} \\ {1.6} & {0 \le Q_{GEN2} < 0.3} \\ 1 & {0.3 \le Q_{GEN2} < 0.5} \\ {8Q_{GEN2} - 2.2} & { - 0.2 \le Q_{GEN2} < 0.12} \\ \end{array} } \right.$$

**Figure 3:**
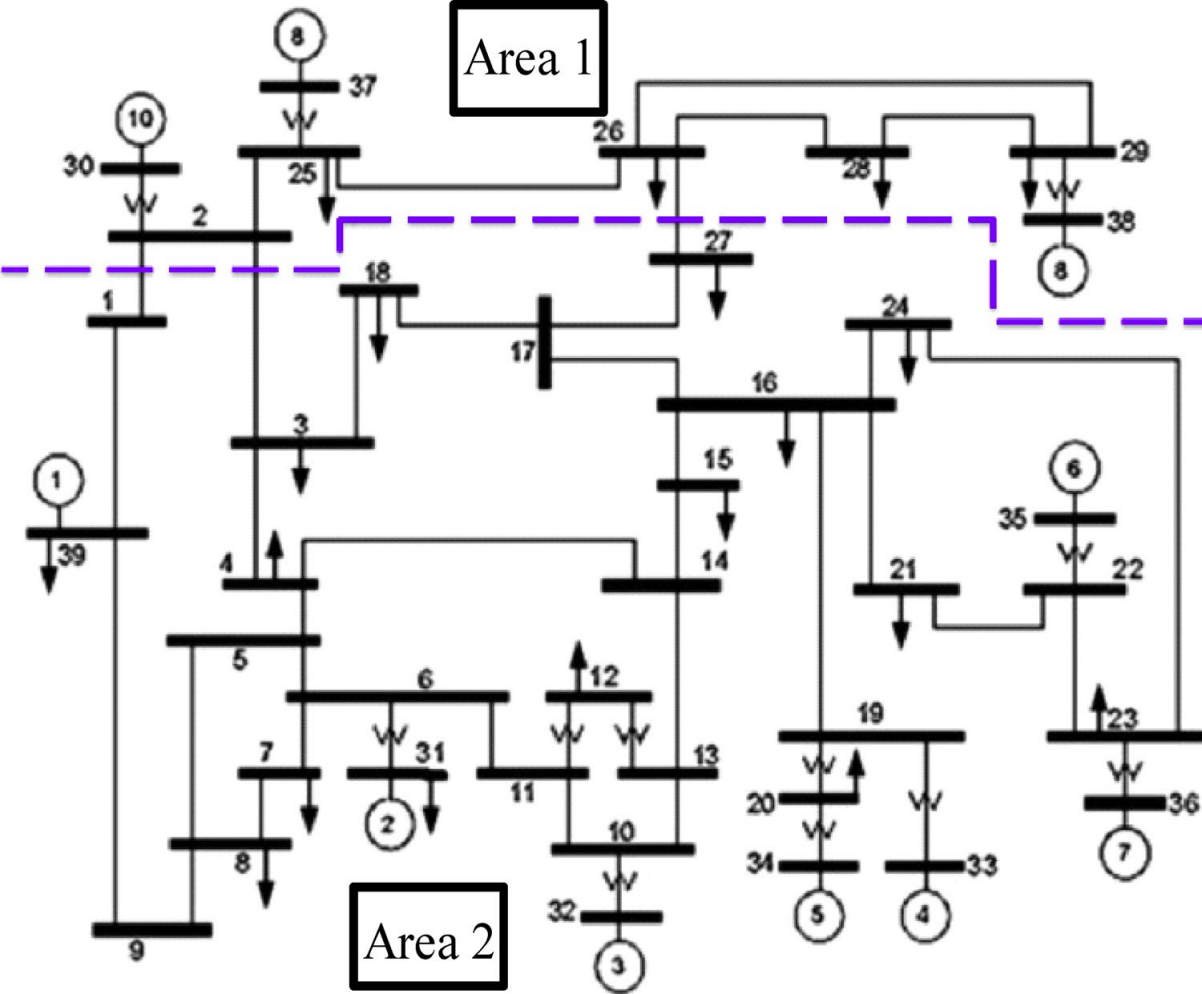
IEEE 39-bus system.

The price per megawatt of production is calculated at $15, with the cost of creating reactive power for static compensators being considered to be fixed. Moreover, the price per unit to replace the transformer's tap is $15. This system has 12 transformers with tap changers, 4 reactive power compensators installed in buses 4, 8, 15, and 27, and 10 generators. The term “slack bus generator” is used to describe the generator linked to bus 31 (see Fig. 3).

This section presents the results obtained from computations conducted using MATLAB software tools. The outcomes of optimization utilizing the Particle Swarm Optimization (PSO) algorithm will be discussed in a subsequent section, accompanied by a comparative analysis against existing methodologies. The analysis commences with the allocation of active power over a specific time period. Initially, generators constitute the sole source of reactive power required for the system. Subsequently, a scenario in which there is an unexpected increase in the total active power demand by a factor of ζ = 0.01 following the optimal allocation of active power to the grid's top is considered. In response to this situation, a decrease in bus voltages is observed, necessitating the provision of reactive power to rectify the voltage reduction. Furthermore, it is assumed that the active power output of the generators, excluding the slack bus generator, remains constant.

Following the resolution of the model, the following conclusions are drawn for both scenarios. The active and reactive power allocations for the generators are detailed in Table 1, while Table 2 presents the reactive power ratings for the static compensators. The transformer tap changer specifications are provided in Table 3, and the quantities and voltage angles of the buses are tabulated in Table 4. Additionally, Table 5 delineates the associated expenditures for each state, along with the supplementary costs incurred in the second state.

**Table 1:** Active and reactive power allocated for generators.

GEN	P_Gen1_ (MW)	P_Gen2_ (MW)	Q_Gen1_ (MVar)	Q_Gen2_ (MVar)
G1	350	350	160.077	− 3.609
G2	242.24	303.11	168.237	28.15
G3	614.46	614.46	199.999	199.99
G4	635.267	635.267	117.079	4.2107
G5	542.254	542.254	173.286	197.54
G6	747.742	747.742	237.701	178.8
G7	581.993	581.993	112.859	199.93
G8	621.277	621.277	11.6495	149.559
G9	762.445	762.445	9.7522	87.888
G10	1100	1100	88.0324	149.98

**Table 2:** Assigned reactive power of the static compensators.

Static compensator	Q_sh1_ (MW)	Q_sh2_ (MW)
Sh1(bus4)	0	25
Sh2(bus8)	0	24.986
Sh3(bus15)	0	17.85
Sh4(bus27)	0	0.010169

**Table 3:** Changeer transformers.

Bstart → Bdestination	Tap1	Tap2
12 → 11	1.006	1.00844
12 → 13	1.006	1.01185
6 → 31	1.07	1.00159
10 → 32	1.07	1.01609
19 → 33	1.07	1.00055
20 → 34	1.009	0.98155
22 → 35	1.025	1.00933
23 → 36	1	1.02094
25 → 37	1.025	1.01279
2 → 30	1.025	0.99885
29 → 38	1.025	1.00577
19 → 20	1.06	1.00217

**Table 4:** Amount and voltage angle of the buses.

BUS	V_bus1(pu)	V_bus2(pu)	Delta_bus1(rad)	Delta_bus2(rad)
B1	1.046	1.0518	− 0.0069	− 0.02881
B2	1.047	1.0452	0.0387	0.018100
B3	1.027	1.0220	− 0.028	− 0.04888
B4	1.0031	0.9896	− 0.0718	− 0.08946
B5	1.005	0.9860	− 0.07039	− 0.08422
B6	1.0081	0.9865	− 0.063	− 0.07553
B7	0.9975	0.9789	− 0.095	− 0.11054
B8	0.99	0.9796	− 0.1012	− 0.11710
B9	1.028	1.0268	− 0.0618	− 0.08123
B10	1.017	0.9993	− 0.0121	− 0.02526
B11	1.012	1.1000	− 0.0292	− 0.04218
B12	1.00016	0.9855	− 0.0258	− 0.03948
B13	1.0142	0.9500	− 0.0201	− 0.03439
B14	1.0110	0.9967	− 0.0379	− 0.05493
B15	1.01323	1.0032	− 0.019	− 0.04108
B16	1.0294	1.0200	0.0161	− 0.00652
B17	1.0315	1.0278	− 0.004	− 0.02759
B18	1.0288	1.0242	− 0.02	− 0.04334
B19	1.048	1.0242	0.103	0.084106
B20	0.99	1.0997	0.083	0.064473
B21	1.0288	1.0197	0.0678	0.045418
B22	1.047	1.0393	0.155	0.134249
B23	1.042	1.0399	0.15	0.128122
B24	1.0347	1.0262	0.0207	− 0.00210
B25	1.0568	1.0687	0.0646	0.033362
B26	1.052	1.0719	0.025	− 0.00383
B27	1.036	1.0459	− 0.008	− 0.03453
B28	1.051	1.0938	0.072	0.037733
B29	1.051	1.0999	0.116	0.077136
B30	1.047	1.1000	0.0979	0.076119
B31#	0.98	0.9500	0	0
B32	0.98	1.1000	0.119	0.098096
B33	0.997	0.9500	0.19	0.170165
B34	1.0123	1.1000	0.180	0.149459
B35	1.0493	1.1000	0.255	0.233393
B36	1.0635	0.9500	0.292	0.274081
B37	1.0278	1.1000	0.2	0.159096
B38	1.0265	0.9500	0.229	0.175011
B39	1.03	1.0392	− 0.034	− 0.05646

**Table 5:** The related costs of each state as well as the additional costs incurred in the second state.

Total cost 1($)	Total cost 2($)	Additional cost($)
38,010.93	44,140.605	6129.675

## Using particle swarm optimization to solve the second mode optimization

This paper initially introduces the proposed methodology employed herein, followed by an elucidation of the Particle Swarm Optimization (PSO) technique. The PSO method, an optimization approach inspired by simulated animal behavior, originated in the 1990s. Specifically, Eberhart and Kennedy pioneered a technique for PSO based on behavioral analogies observed in fish and birds. A notable characteristic of this method is its capacity to operate based on relatively simplistic rules.

The underlying principle of the PSO technique revolves around the concept that individuals base their decisions on two types of information: personal experiences and observations of others' experiences. Individuals explore various options to discern their relative superiority and desirability, drawing upon their own past encounters. Additionally, they also consider the behaviors and outcomes of those in their proximity, leveraging external experiences to inform their decision-making process. Therefore, using the following information, each particle strives to enhance its position:

- Vector of the current position
- Current velocity vector
- The distance between the current position and the most advantageous position encountered
- The separation between the present position and the particle swarm's optimal position

The following equations can be used to formulate position adjustment, in accordance with the aforementioned sentences:

3 $$\begin{aligned} V_{i}^{N + 1} &= MV_{i}^{N} + J_{1} rand_{1} \times (pbest_{i} - Z_{i}^{N} ) \hfill \\ & \quad + J_{2} rand_{2} \times (gbest - Z_{i}^{N} ) \hfill \\ \end{aligned}$$

and

4 $$M = M_{\max } - \frac{{M_{\max } -M_{\min } }}{{iter_{\max } }} \times iter$$

5 $$Z_{i}^{N + 1} = Z_{i}^{N} + V_{i}^{N + 1}$$

where $z_{i}^{N}$ is the current position of the i-th particle in the k-th iteration, $pbest_{i}$ is the best position the i-th part has ever experienced, and gbest is the best experienced position of the particle swarm. $V_{i}^{N}$ is the velocity vector of the i-th particle in the k-iteration, w is the weighting function, $J_{j}$ is the weighting coefficient, and rand is the random vector of particle i between zero and one. Additionally, $M_{\max }$ and $M_{\min }$ are the highest and lowest weights, respectively, $iter_{\max }$ is the number of repetitions, and $iter$ is the current repetition.

**Figure 4:**
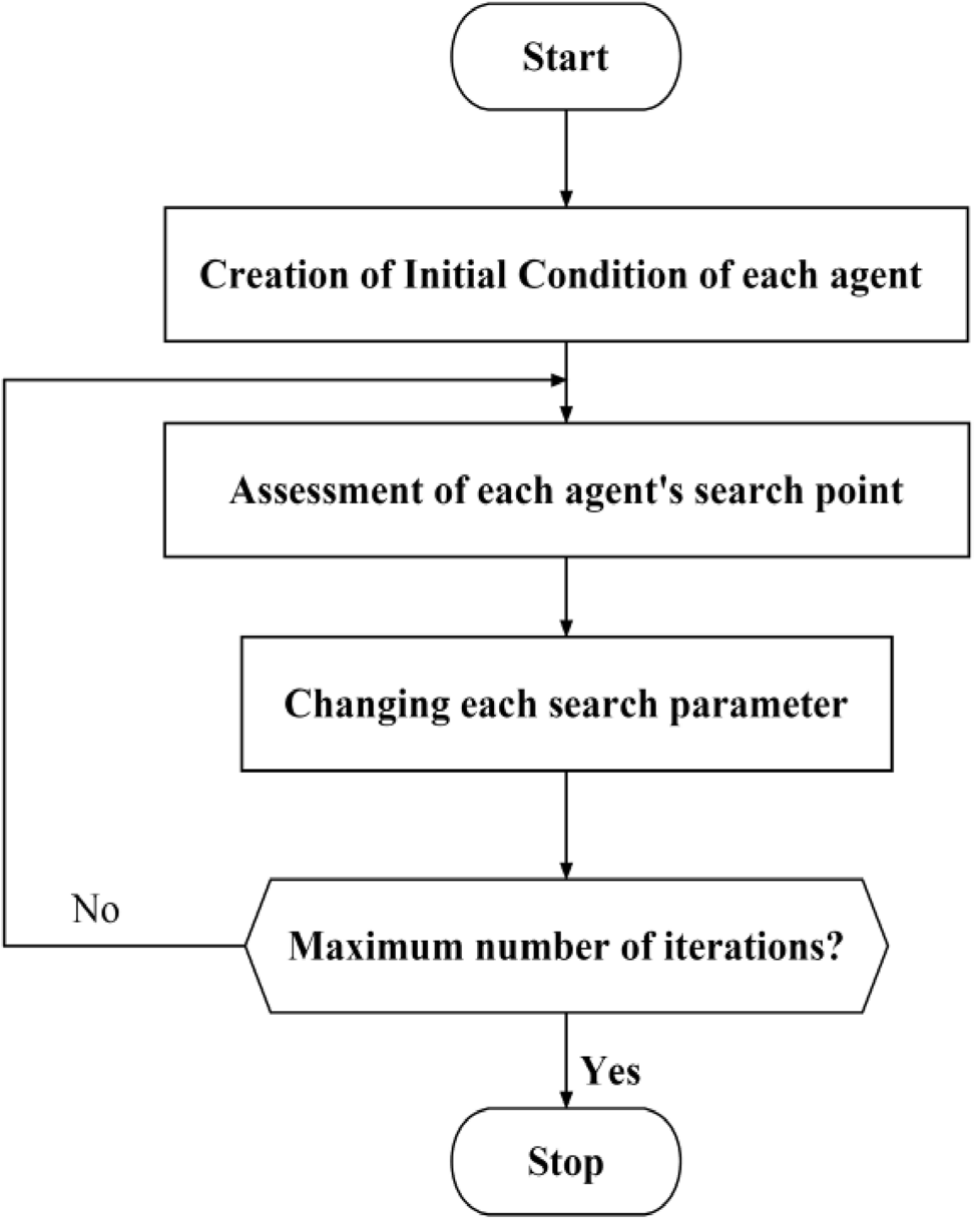
General process of particle swarm optimization.

Experience has demonstrated that the following values $M_{\min } = 0.4$ and $$\begin{array}{*{20}c} {M_{\max } = 0.4} & {J_{j} } \\ \end{array} = 2$$ for these parameters are appropriate for power system issues. The main advantages of this technique are shown in Fig. 4:

This research proposes an approach that surpasses customary methods in both accuracy and speed. Specifically, if a particle's current position in each iteration proves to be inferior to its best-experienced position, an adjustment is made to its velocity vector. This adjustment aims to propel the particle's future position away from its current location, thereby averting movement toward unfavorable conditions in subsequent cycles. This adjustment is mathematically expressed as follows:

6 $$\begin{aligned} V_{i}^{N + 1} &= MV_{i}^{N} + J_{1} rand_{1} \times (pbest_{i} - Z_{i}^{N} ) \hfill \\&\quad + J_{2} rand_{2} \times (gbest - Z_{i}^{N} ) + J_{3} rand_{3} \times (Z_{i}^{N} - pworse_{i} ) \hfill \\ \end{aligned}$$

where $pworse_{i}$ is the place prior to the I particle that has poor experience. To address the optimal distribution of reactive power during operation, it is essential to define the variable vector for each particle. The grid under consideration encompasses 39 buses and 12 transformers equipped with tap changers, resulting in a total of 90 entries for each particle's variable vector. Specifically, the first 39 entries pertain to the bus voltage magnitude, the subsequent 39 entries correspond to the bus voltage phase angle, and the final 12 entries are associated with the tap settings of the transformers.

**Figure 5:**
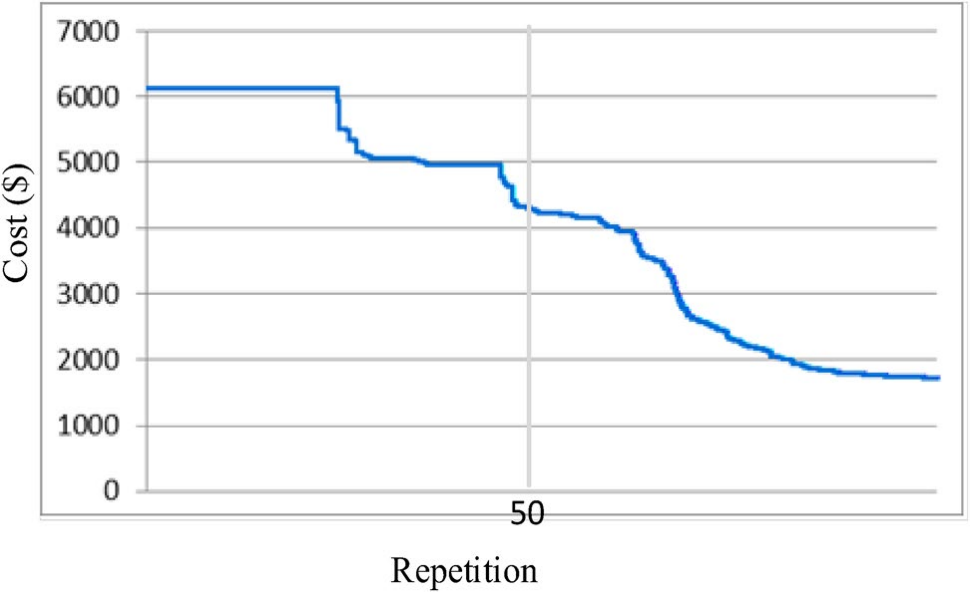
Amount of objective function (additional cost in dollars) during 100 repetitions.

In this optimization strategy, 200 particles were employed, and 100 iterations were conducted to ensure convergence. The optimization outcomes achieved through this method are illustrated in Fig. 5, which also displays the power generated by generators before and after a significant increase in load. The transformer tap changer settings are provided in Table 6, while Table 7 outlines the allocated reactive power for the static compensators. Tables 8 and 9 present the quantities and voltage angles of the buses. Furthermore, Table 10 provides details on the expenditures associated with each mode, including the additional costs incurred in the second mode.

**Table 6:** Amount of active and reactive power generated by generators before and after sudden increase in load.

GEN	PGen1 (MW)	PGen2 PSO (MW)	QGen1 (MVar)	QGen3 PSO (MVar)
G1	350	350.162	160.077	21.872
G2	242.24	303.158	168.237	30.058
G3	614.46	614.690	199.999	200.00
G4	635.267	637.043	117.079	30.202
G5	542.254	541.863	173.286	173.35
G6	747.742	747.630	237.701	237.72
G7	581.993	579.594	112.859	112.94
G8	621.277	621.048	11.6495	50.154
G9	762.445	762.432	9.7522	53.115
G10	1100	1100.66	88.0324	89.020

**Table 7:** Changeer transformers.

Bstart → Bdestination	Tap1	Tap2_PSO
12 → 11	1.006	1.008
12 → 13	1.006	1.011
6 → 31	1.07	1.001
10 → 32	1.07	1.016
19 → 33	1.07	1.002
20 → 34	1.009	0.980
22 → 35	1.025	1.013
23 → 36	1	1.010
25 → 37	1.025	1.002
2 → 30	1.025	1.000
29 → 38	1.025	1.003
19 → 20	1.06	1.001

**Table 8:** Assigned reactive power of the static compensators.

Static compensator	Qsh1	Qsh2_PSO
SH1 (bus4)	0	− 0.00724
SH2 (bus8)	0	0.026266
SH3 (bus15)	0	− 0.01454
SH4 (bus27)	0	0.045428

**Table 9:** Amount and voltage angle of buses.

BUS	V_bus1 (pu)	V_bus2_PSO (pu)	Delta_bus1 (rad)	Delta_bus2_PSO (rad)
B1	1.046	1.04782	− 0.0069	− 0.0285
B2	1.047	1.04557	0.0387	0.01788
B3	1.027	1.02199	− 0.028	− 0.0489
B4	1.0031	0.98828	− 0.0718	− 0.0893
B5	1.005	0.98589	− 0.07039	− 0.0842
B6	1.0081	0.98665	− 0.063	− 0.0755
B7	0.9975	0.97892	− 0.095	− 0.1105
B8	0.99	0.97901	− 0.1012	− 0.1170
B9	1.028	1.02968	− 0.0618	− 0.0815
B10	1.017	0.99934	− 0.0121	− 0.0252
B11	1.012	1.1	− 0.0292	− 0.0422
B12	1.00016	0.98547	− 0.0258	− 0.0395
B13	1.0142	0.95	− 0.0201	− 0.0344
B14	1.0110	0.99657	− 0.0379	− 0.0548
B15	1.01323	1.00205	− 0.019	− 0.0408
B16	1.0294	1.02000	0.0161	− 0.0064
B17	1.0315	1.02780	− 0.004	− 0.0275
B18	1.0288	1.02422	− 0.02	− 0.0433
B19	1.048	1.02720	0.103	0.08396
B20	0.99	1.09999	0.083	0.06447
B21	1.0288	1.02004	0.0678	0.04534
B22	1.047	1.03965	0.155	0.13401
B23	1.042	1.03502	0.15	0.12811
B24	1.0347	1.02598	0.0207	− 0.0021
B25	1.0568	1.06801	0.0646	0.03376
B26	1.052	1.07176	0.025	− 0.0038
B27	1.036	1.04582	− 0.008	− 0.0345
B28	1.051	1.09410	0.072	0.03770
B29	1.051	1.1	0.116	0.07712
B30	1.047	1.1	0.0979	0.07589
B31#	0.98	0.95	0	0
B32	0.98	1.1	0.119	0.09813
B33	0.997	0.95	0.19	0.16959
B34	1.0123	1.1	0.180	0.14939
B35	1.0493	1.1	0.255	0.23308
B36	1.0635	0.95	0.292	0.27527
B37	1.0278	1.1	0.2	0.16015
B38	1.0265	0.95	0.229	0.17522
B39	1.03	1.03106	− 0.034	− 0.0561

**Table 10:** Costs corresponding to each mode and additional costs imposed in the second mode.

Total cost 1 ($)	Total cost 2_PSO ($)	Additional cost ($)
38,010.93	39,739.0635	1728.1335

**Figure 6:**
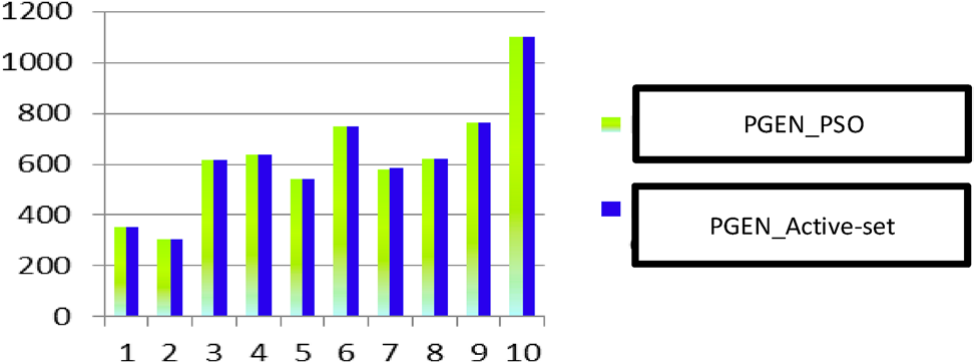
Comparison of two optimization techniques in terms of the active generating power of generators.

**Figure 7:**
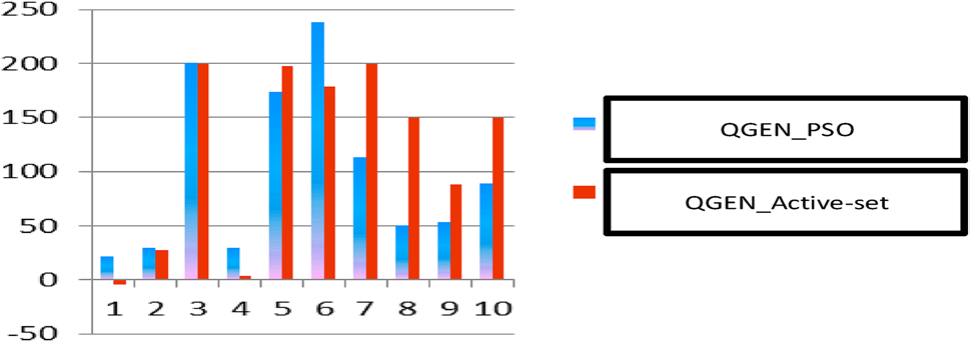
Comparison of two methods for optimizing the reactive power generated by generators.

**Figure 8:**
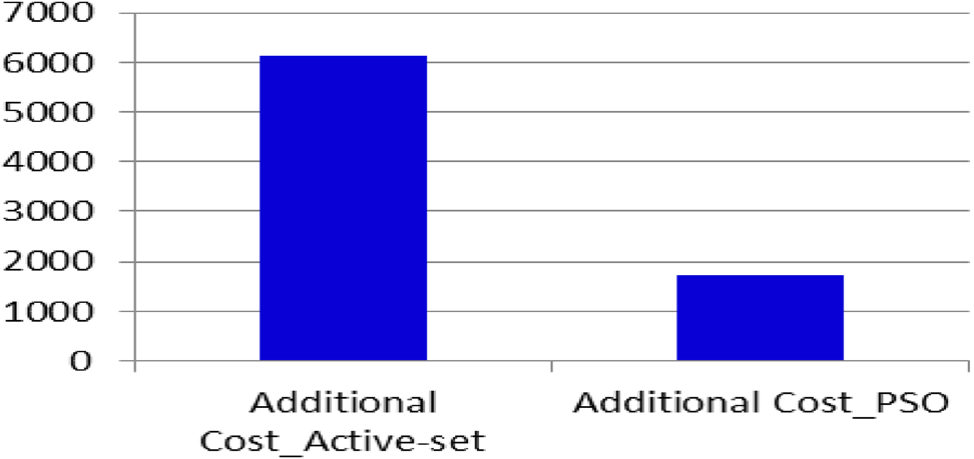
The additional cost of a sudden increase in load in the two methods.

The active generating power of generators using two optimization techniques is compared in Fig. 6. In Fig. 7, two approaches to maximizing the reactive power produced by generators are compared. Figure 8 An unexpected increase in the load's additional cost using two different techniques.

## Conclusions and discussion

Reactive power plays crucial roles in power system reliability and security. Market participants utilize the network differently to maximize their profits. This means that their effects on the system, such as losses, can also be different. The development of a fair and accurate loss allocation scheme for real and reactive power is important for avoiding cross subsidies and obtaining the correct charge for each participant. To minimize costs, this paper introduces a model for the optimal allocation of reactive power. Technically, this model utilizes the voltage stability margin as a safeguard for operational safety and ensures the attainment of maximum active power contracts in the market from an economic standpoint. Reactive power provision is regarded as an additional service within this framework. The efficacy of the proposed model has been evaluated using the 39-bus IEEE system, and simulation results employing the particle swarm optimization algorithm showcase its ability to achieve optimal reactive power allocation during operation, notwithstanding the constraints of the market environment.

## Data Availability

The data can be shared upon request to the corresponding author.
